# Complete Genome Sequence of *Vibrio campbellii* LMB 29 Isolated from Red Drum with Four Native Megaplasmids

**DOI:** 10.3389/fmicb.2017.02035

**Published:** 2017-10-23

**Authors:** Jinxin Liu, Zhe Zhao, Yiqing Deng, Yan Shi, Yupeng Liu, Chao Wu, Peng Luo, Chaoqun Hu

**Affiliations:** ^1^Institute of Marine Biology, College of Oceanography, Hohai University, Nanjing, China; ^2^Department of Food Science and Technology, University of California, Davis, Davis, CA, United States; ^3^Key Laboratory of Tropical Marine Bio-resources and Ecology, Guangdong Provincial Key Laboratory of Applied Marine Biology, South China Sea Institute of Oceanology, Chinese Academy of Sciences, Guangzhou, China

**Keywords:** *Vibrio campbellii*, whole genome sequencing, T6SS, rifampicin resistance, *arr-9*, virulence, *tlh*

## Abstract

*Vibrio* spp. are the most common pathogens for animals reared in aquaculture. *Vibrio campbellii*, which is often involved in shrimp, fish and mollusks diseases, is widely distributed in the marine environment worldwide, but our knowledge about its pathogenesis and antimicrobial resistance is very limited. The existence of this knowledge gap is at least partially because that *V. campbellii* was originally classified as *Vibrio harveyi*, and the detailed information of its comparative genome analysis to other *Vibrio* spp. is currently lacking. In this study, the complete genome of a *V. campbellii* predominant strain, LMB29, was determined by MiSeq in conjunction with PacBio SMRT sequencing. This genome consists of two circular DNA chromosomes and four megaplasmids. Comparative genome analysis indicates that LMB29 shares a 96.66% similarity (average nucleotide identity) with the *V. campbellii* ATCC strain BAA-1116 based on a 75% AF (average fraction) calculations, and its functional profile is very similar to *V. campbellii* E1 and *V. campbellii* CAIM115. Both type III secretion system (T3SS) and type VI secretion system (T6SS), along with the *tlh* gene which encodes a thermolabile hemolysin, are present in LMB29 which may contribute to the bacterial pathogenesis. The virulence of this strain was experimental confirmed by performing a LDH assay on a fish cell infection model, and cell death was observed as early as within 3 h post infection. Thirty-seven antimicrobial resistance genes (>45% identity) were predicted in LMB29 which includes a novel rifampicin ADP ribosyltransferase, *arr-9*, in plasmid pLMB157. The gene *arr*-9 was predicted on a genomic island with horizontal transferable potentials which may facilitate the rifampicin resistance dissemination. Future researches are needed to explore the pathogenesis of *V. campbellii* LMB29, but the availability of this genome sequence will certainly aid as a basis for further analysis.

## Introduction

*Vibrio campbellii* is a Gram-negative bacterium that has been found widely distributed in marine associated aquatic environments (Thompson et al., 2004). This bacterium was once classified as non-pathogenic, non-luminescent *Vibrio* spp. probably because it was misidentified with *Vibrio harveyi* for years (de la Pena et al., 2001; Lin et al., 2010). Indeed, studies indicate this bacterium is an important pathogen that contributes to luminescent disease in farmed shrimp (Gomez-Gil et al., 2004; Phuoc et al., 2008; Wang L. P. et al., 2015), causes mortality in reared fish and shellfish (Thompson et al., 2004), and may also be involved in the process of coral bleaching (Thompson et al., 2005).

The red drum (*Sciaenops ocellatus*), also known as channel bass, spottail bass, or redfish, is originally distributed in the Atlantic Ocean and the Gulf of Mexico. This species is commercially valuable sciaenid with demonstrated suitability for mariculture, and has become one of important cage-aquaculture fish since its introduction into China in 1995 (Shao, 2010). Vibriosis are a major obstacle for healthy and sustainable development of mariculture in China. One predominant strain *V. campbellii* LMB29, with a multi-drug resistance phenotype (ampicillin, amoxicillin, rifampicin, tetracycline, streptomycin, oxacillin, lincomycin, teicoplanin, vancomycin), was isolated from a cage-cultured red drum in Shenzhen, China (2012) and was suspected to contribute to serious skin ulcer of the fish. Scientific researches are needed to study its pathogenesis to reduce the economic losses.

In this study, we characterized the complete genome sequences of *V. campbellii* strain LMB29 to investigate its virulence and antimicrobial resistance. Multiple virulence factors were identified in this strain, including a gene encoding the thermolabile hemolysin (*tlh*) which is normally considered a significant molecular marker for pathogenic *Vibrio parahaemolyticus* (Gutierrez West et al., 2013). In addition to type III secretion system (T3SS), a recently characterized type VI secretion system (T6SS) (Ho et al., 2014) is also present in this strain which suggests that LMB29 is virulent. Its virulence was further confirmed by using a LDH assay on a fish cell infection model. Thirty-seven antimicrobial resistance genes (>45% identity) were predicted in this strain which includes a novel rifampicinn ADP ribosyltransferase, *arr-9*, in plasmid pLMB157. The gene *arr*-9 was predicted on a genomic island and it is the first report about this gene in a transmissible plasmid from *Vibrio* spp. The availability of this genome sequences along with our preliminary findings of *V. campbellii* LMB29 will aid as a basis for further analysis of pathogenesis and antimicrobial resistance of *Vibrio* species in marine aquaculture system.

## Materials and methods

### Genome DNA preparation and whole genome sequencing

*Vibrio campbellii* LMB29 was isolated from skin ulcer sample of cage-cultured red drum in Shenzhen, China by 2012. Briefly, diseased tissue mixtures were plated onto thiosulphate citrate bile salts sucrose (TCBS) agar for incubation under 30°C. 24–36 h later, morphologically uniform colonies of *Vibrio* were passaged onto fresh media to obtain pure colonies. The strain identity of individual isolate was confirmed with optimized multilocus sequence analysis (MLSA) (Gabriel et al., 2014). The predominant strain, *V. campbellii* LMB29, was grown in TSB media at 30°C and its genomic DNA was extracted by using the Cetyltrimethyl Ammonium Bromide (CTAB) method with minor modification (Healey et al., 2014). The quantity and quality of genomic DNA were assessed by using a Qubit 2.0 Fluorometer (Thermo Scientific, USA) and a NanoDrop 2000 Spectrophotometer (Thermo Scientific, USA), respectively. The whole genome sequencing was performed by a commercial vendor (Personalbio, Shanghai, China) by using Illumina MiSeq (400 bp inserts library with 251 bp paired-end sequencing; Illumina, San Diego, CA, USA) and PacBio RS II platform (10 kb inserts library; Pacific Biosciences, Menlo Park, CA, USA).

### Genome assembly and annotation

The trimmed sequences from Illumina MiSeq were assembled using Newbler version 2.8 (454 Life Sciences, Branford, CT, USA) and PacBio sequencing reads were *de novo* assembled with hierarchical genome-assembly process (HGAP) (Chin et al., 2013). Sequencing data were combined, and further polished with Pion (Walker et al., 2014) to obtain the complete sequences. The prediction of open reading frames (ORFs) and their annotations were performed using Glimmer 3.0 (Delcher et al., 2007). The genome annotation was further corrected and confirmed with the NCBI Prokaryotic Genome Automatic Annotation Pipeline (PGAAP) (Angiuoli et al., 2008) and DOE-JGI Microbial Genome Annotation Pipeline (Huntemann et al., 2015). The putative virulence factors of the *V. campbellii* LMB29 strain were predicted using the basic local alignment search tool (BLAST) in the Virulence Factor Database (VFDB; http://www.mgc.ac.cn/VFs/main.htm) (Chen et al., 2016). Contigs were subsequently queried with Resfinder (Zankari et al., 2012) and CARD (McArthur et al., 2013) to perform antimicrobial resistance genes analysis.

### Comparative genome analysis

The strain was originally identified as *V. campbellii* based on 16S rRNA sequencing and its identity was further confirmed with comparative phylogenetic tree analysis using MEGA7 (Kumar et al., 2016) with various 16S rRNA sequences of the genus *Vibrio*. The phylogenetic tree was constructed from the aligned sequences using Kimura 2-parameter model with the neighbor-joining method, bootstrapped 1,000 times via MEGA7 software. The *V. campbellii* LMB29 assembled contigs were also submitted to JGI IMG/MER database (Chen et al., 2017) (https://img.jgi.doe.gov/cgi-bin/mer/main.cgi) to perform the Average Nucleotide Identity (ANI) calculations (IMG Genome ID: 2718217691). In addition, 1,045 *Vibrio* complete genomes (Supplementary Table 1) were retrieved from JGI (all available *Vibrio* genome in JGI) to perform genome comparisons (**Figure 9**) and functional profile analysis (**Figure 10**) based on the Clusters of Orthologous Groups (COGs) database (Tatusov et al., 2000). Bacterial genome, plasmids and ICEs sequences alignments were performed using Mauve with progressive Mauve option (Darling et al., 2004). IslandViewer 4 was applied to predict genomic islands in *V. campbellii* LMB29 with default settings (Bertelli et al., 2017). DEG (Database of Essential Genes) database (version 14.7) was used to assess gene essentiality of ORFs predicted in LMB29 with BLAST search (Luo et al., 2014).

### Infection and lactate dehydrogenase (LDH) assay

Fathead minnow (FHM) epithelial cells were routinely cultured in M199 medium supplemented with 10% (v/v) fetal bovine serum (FBS, Gibco) at 28°C. For LDH assays, FHM cells were seeded into 96-well plates and grown overnight to 90% confluence of cell monolayers. Prior to infection, growth media was replaced by 110 μL of serum-free TC199 medium per well, and cells were infected with *V. campbellii* LMB29 at a multiplicity of infection (MOI) of 5 over a 6 h time course. At the indicated time point, the 96-well plates were centrifuged at 3,200 × g for 2 min, and a 100 μl aliquot of the supernatant was removed for measuring LDH release with the Cytotoxicity Detection kit^PLUS^ per manufacturer's instructions (Roche).

### Construction of deletion mutant and complementation

The deletion of *arr*-9 gene was performed by allelic exchange. Briefly, primers pSW7848-F/R were used to linearize the suicide vector pSW7848, and the primers Arr9-up-F/R and Arr9-down-F/R (Supplementary Table 2) were used to amplify two fragments flanking the coding sequence of *arr-*9. The two fragments, respectively incorporated 18 and 17 bp overlapping sequences with vector pSW7848, which also contained 17 bp overlapping sequences between them. The linearized vector and flanking fragments were assembled according to the instruction of the ClonExpress MultiS One Step Cloning Kit (Vazyme Biotech, China), generating pSW7848_Δ*arr*-9. The resultant plasmid was transformed into chemical competent cells of *E. coli* GEB883 and then transferred into *V. campbellii* LMB29 by conjugation. The *arr-*9 deletion strain (LMB29Δ*arr*-9) was selected on TCBS plates containing 20 μg/mL chloramphenicol and 0.2% glucose followed by a 0.2% arabinose selection process. Gene deletion was then confirmed by PCR and sequencing with primers Arr9-outer-F/R.

To complement the *arr-*9 gene, primers pMMB207-F/R were used to linearize the expression vector pMMB207 and primers Arr9-F/R were used to amplify the complete *arr-*9, including the ORF, the promoter and the terminator. The resulting *arr-*9 amplicon was assembled into the linearized expression vector pMMB207 by using the ClonExpress II One Step Cloning Kit (Vazyme Biotech, China). The recombinant pMMB207_*arr*-9 plasmid was respectively introduced by conjugation into the *arr-*9 deletion strain and wild type LMB29, resulting in LMB29Δ*arr*-9:pMMB207_*arr*-9 and LMB29:pMMB207_*arr*-9. The empty pMMB207 plasmid was included as a control.

### Rifampicin susceptibility assay

Rifampicin susceptibility of the wild type LMB29, the *arr-*9 deletion mutant and the complementation strain were tested with the *E*-test (BioMerieux SA, France) according to the manufacturer's guidance. Briefly, overnight cultures were diluted 1:25 into fresh TSB, and each 20 μL of dilution was then plated on TSB plates using sterile swab. The antibiotic strips were individually placed on the dried plates and the Minimum Inhibitory Concentration (MIC) was determined after an incubation of 48 h at 30°C. The MIC was interpreted the value at which the elliptical inhibition zone intercepted the scale on the *E*-test strip.

## Results

### Genome characteristics

**Figure 1:**
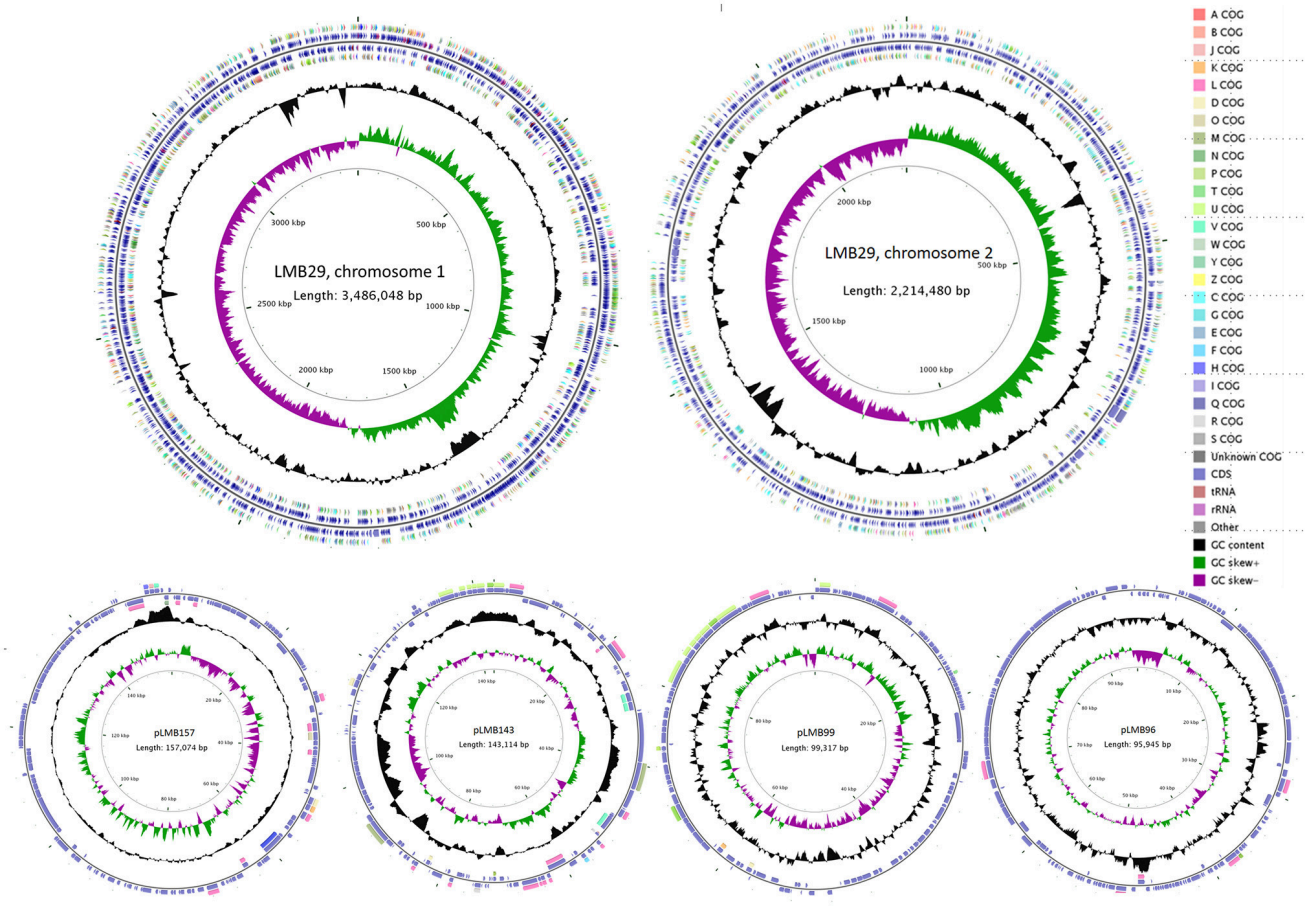
The circular maps of the *V. campbellii* LMB29. Coding sequences (CDS) were colored into COG family proteins.

A total of 2,689,618 high-quality paired reads of 634,307,780 clean bases were generated through the Illumina MiSeq pipeline which represents a 102X sequencing coverage. The PacBio SMRT sequencing, with a N50 of 7,484 bp, was also introduced to close the gaps among assembled contigs. Combined sequencing analysis revealed that the complete genome of *V. campbellii* LMB29 includes two circular DNA chromosomes and four megaplasmids. Chromosome I consists of 3,486,048 bp with a GC content of 45.54% containing 3,072 predicted ORFs, 110 tRNAs and 34 rRNAs. Chromosome II consists of 2,214,480 bp with a GC content of 45.23% containing 2,004 predicted ORFs, 15 tRNAs and 3 rRNAs. Plasmid pLMB157 consists of 157,074 bp with a GC content of 40.37% containing 160 predicted ORFs. Plasmid pLMB143 consists of 143,114 bp with a GC content of 42.30% containing 165 predicted ORFs, and 2 tRNAs. Plasmid pLMB99 consists of 99,317 bp with a GC content of 39.57% containing 146 predicted ORFs. Plasmid pLMB96 consists of 95,945 bp with a GC content of 40.76% containing 148 predicted ORFs. Circular genome maps were generated for individual chromosome and plasmid based on bioinformatics analysis (Figure 1).

**Figure 2:**
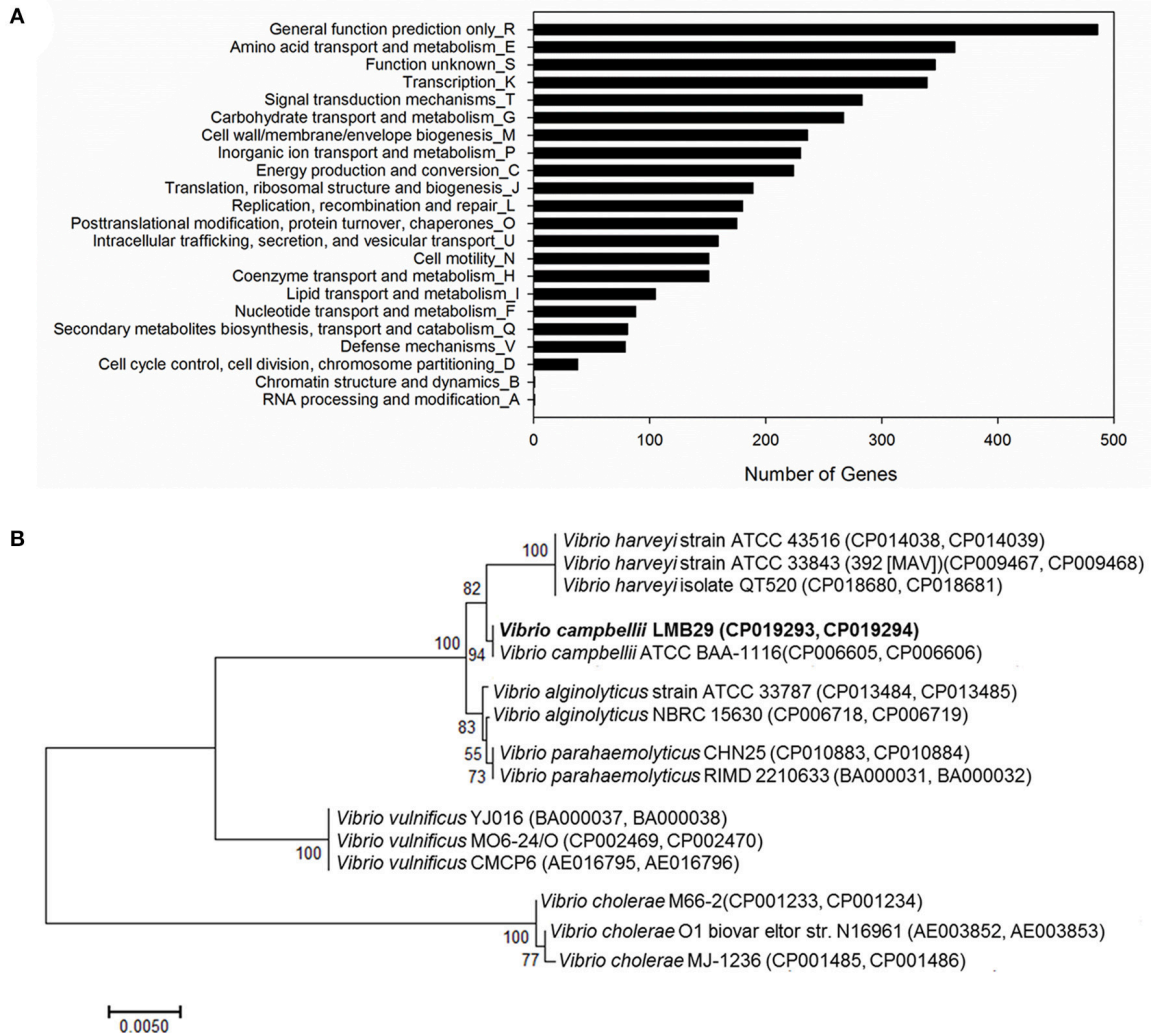
**(A)** Functional categorization of *V. campbellii* LMB29 based on the COG database. **(B)** The phylogenetic tree of the *V. campbellii* LMB29 with closely related *Vibrio* species aligned by using ClustalW.

The predicted ORFs are further classified into COGs functional groups (2,154 ORFs in total; Figure 2A). According to the COG categorization analysis, based on gene counts, the abundant groups include class of R (486 ORFs, general function prediction only), class of E (363 ORFs, amino acid transport and metabolism), class of S (346 ORFs, function unknown), class of K (339 ORFs, transcription), and class of T (283 ORFs, signal transduction mechanisms; Figure 2A). The genomic sequences were used to against the DEG database to perform gene essentiality analysis. A total of 2,871 ORFs were predicted as essential genes in *V. campbellii* LMB29 with multiple genes were predicted on genomic islands with horizontal transferability (Supplementary Table 3).

**Figure 3:**
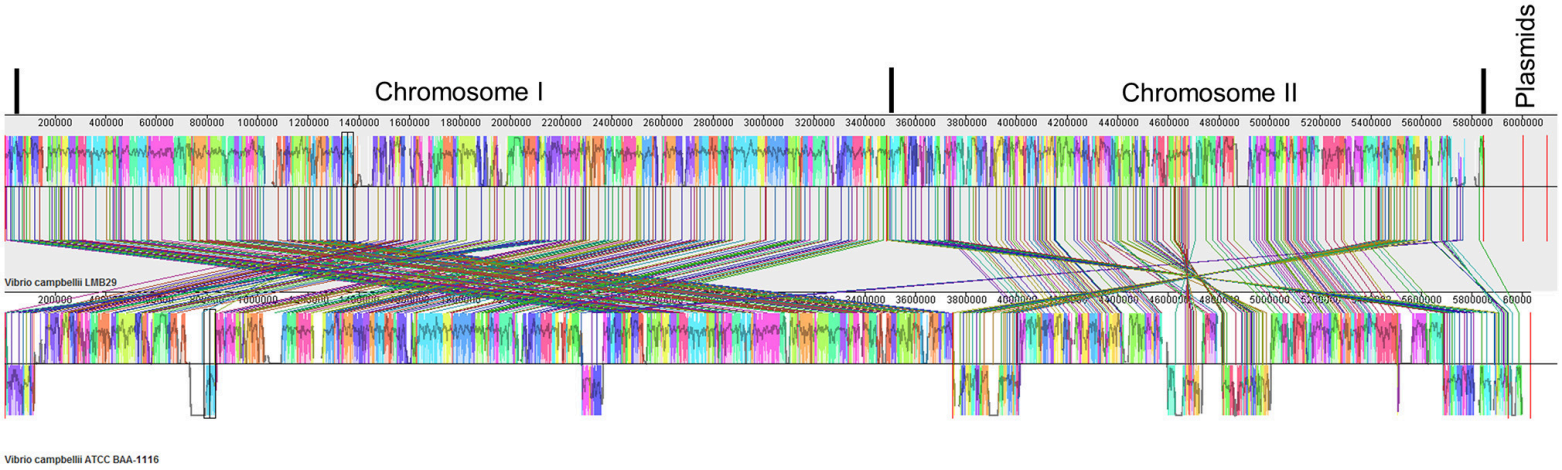
Genome alignments for *V. campbellii* LMB 29 and *V. campbellii* ATCC BAA-1116 using Mauve. Color blocks in the first genome (*V. campbellii* LMB29) are connected by lines to similarly colored blocks in the second genome (*V. campbellii* ATCC BAA-1116). When a block lies above the center line the aligned region is in the forward orientation relative to the first genome sequence. Blocks below the center line indicate regions that align in the reverse complement (inverse) orientation. Regions outside blocks lack detectable homology among the input genomes. Inside each block Mauve draws a similarity profile of the genome sequence. The height of the similarity profile corresponds to the average level of conservation in that region of the genome sequence.

Genome tree analysis was performed on 15 *Vibrio* species with 16S rRNA sequences alignment and strain LMB29 shows very close phylogenetic distance to *V. campbellii* ATCC BAA-1116 (Figure 2B). ANI values indicate that strain LMB29 shares a 96.66% similarity with the ATCC strain BAA-1116 based on a 75% AF (average fraction) calculations, but multiple gene rearrangements also happened in the chromosomes (Figure 3). Compared to the single plasmid (~89 kb; NC_022271.1) in *V. campbellii* ATCC BAA-1116, the *V. campbellii* LMB29 has a unique plasmid profile with four megaplasmids (Figure 3) which indicates the bacterial evolution of *V. campbellii*.

### Virulence and antimicrobial resistance genes

**Figure 4:**
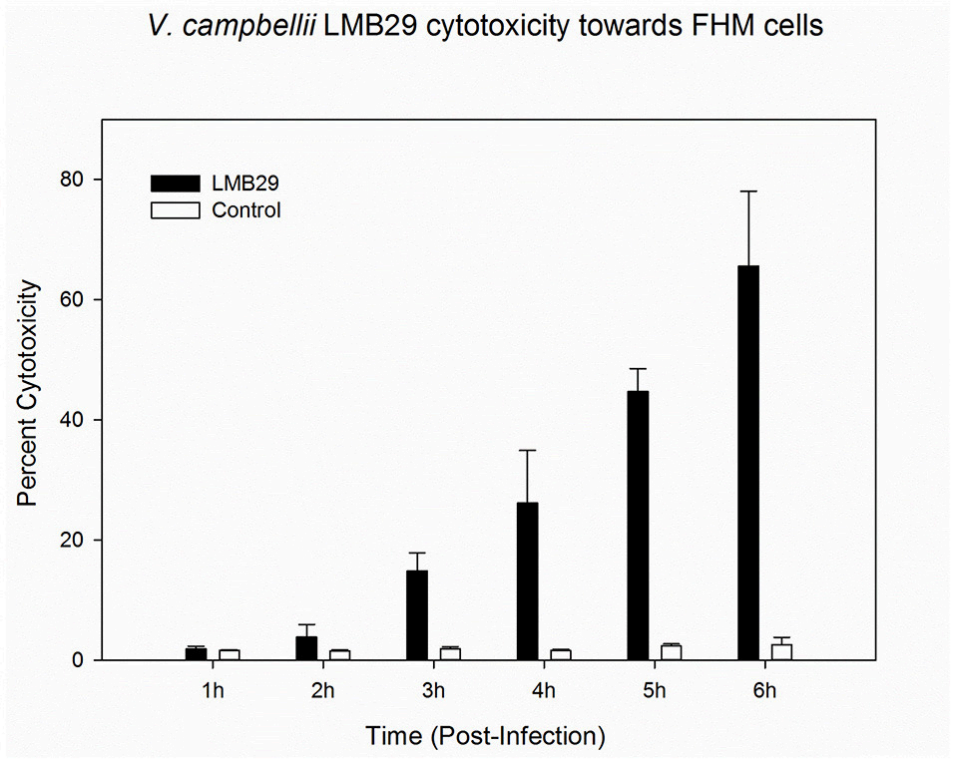
*V. campbellii* LMB 29 cytotoxicity toward FHM cells. FHM cells were infected with LMB 29 strain or uninfected as described in the Materials and Methods. At the indicated time points, culture supernatants were measured for the release of LDH and calculated as a percentage of total cellular lysis. The data are expressed as means ± SEM from three independent experiments.

**Figure 5:**
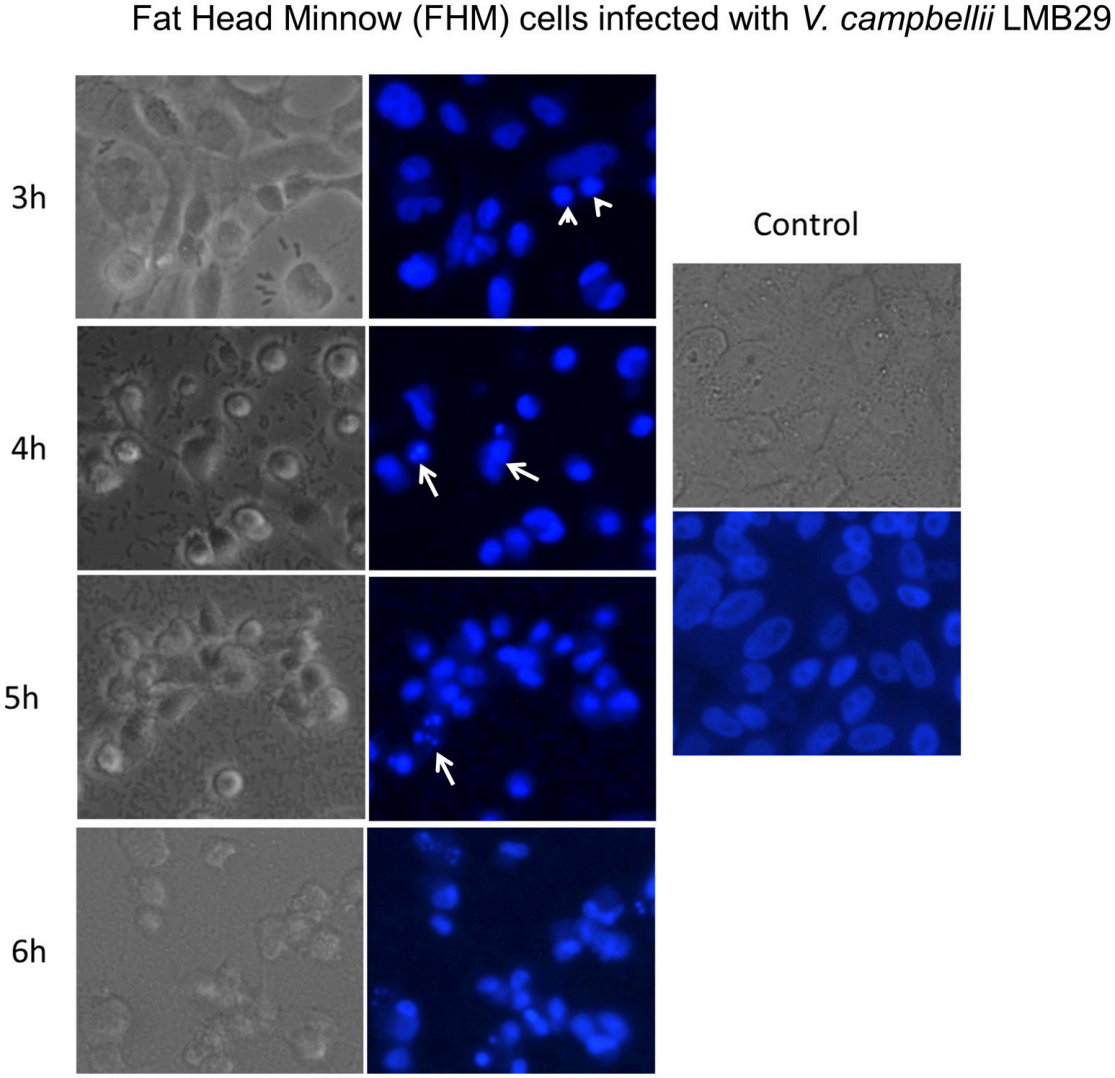
*V. campbellii* LMB 29 infection induces nuclear fragmentation in FHM cells. FHM cells infected with LMB29 strain or uninfected as described in the Materials and Methods, were stained with Hoechst33258 at indicated time points post-infection. The fluorescence signal was detected under an inverted fluorescence microscope (Leica). Arrows indicate the fragmented nuclei, while arrowheads indicate condensed nuclei.

The putative virulence factors in *V. campbellii* LMB29 were analyzed by inquiring the VFDB database (Chen et al., 2016; Table 1). T3SS, which is usually involved in *Vibrio* pathogenesis, was found on chromosome I. Importantly, the recently identified T6SS (Coulthurst, 2013; Ho et al., 2014) was predicted in strain LMB 29 with T6SS1 (1,649,571–1,672,337 region) on chromosome I, T6SS2 (850,171–881,172 region) and T6SS3 (936,359–959,932 region) on chromosome II (Table 1). T6SSs have been implicated in eukaryotic cell targeting and virulence (Coulthurst, 2013; Ho et al., 2014), and is also believed to have anti-bacterial properties in *Vibrio* (Church et al., 2016). In addition, the *tlh* gene encoding a thermolabile hemolysin (TLH) of *V. parahaemolyticus* was found on chromosome II in strain LMB29. TLH is expressed by all clinical and environmental strains of *V. parahaemolyticus* (Bej et al., 1999) and it may have similar biological functions similar to these of the TDH (thermostable direct hemolysin) and TRH (TDH-related hemolysin) toxins, playing a key role in *Vibrio* infections (Wang R. et al., 2015). We also performed LDH release assay using FHM cells (Liu et al., 2016) to test the cytotoxicity activity (Figure 4). Cell death was observed after 3 h post-infection and up to 80% of the cells are lysed within 6 h (Figure 4). Nuclear condensation and fragmentation, based on one DNA staining assay, was confirmed to contribute to the cell death after the infection with *V. campbellii* LMB29 (Figure 5). Taken together, we conclude here that *V. campbellii* strain LMB29 is pathogenic which is consistent with our clinical observations in Shenzhen, China.

**Table 1:** Virulence factors of *Vibrio campbellii* LMB 29.

**Virulence factor**	**Annotation**	**Chromosome**	**Location**
**ADHERENCE**			
*mshI, mshJ, mshL, mshM, mshN, mshE, mshG, mshA, mshC, mshD, mshO, mshP, mshQ*	Mannose-sensitive hemaglutinin (MSHA type IV pilus)	Chromosome I	369152–386356
*pilB*	Type IV pilus	Chromosome I	563010–564695
**ANTIPHAGOZYTOSIS**			
*wza*	Capsular polysaccharide	Chromosome II	262050–3168
*flaG, fliD, fliT, fliS, fliE, fliF, fliG, fliH, fliJ, fliK, fliL, fliM, fliN, fliO, fliP, fliQ, fliR, flhB, flhA, flhF, flgM, flgK, flgJ, flgI, flgF, flgE, flgD, flgC, flgB, flgA, flgM*	Flagella	Chromosome I	885234–81164 2707461–24810
*motY, motB, motA*	Flagella motor protein	Chromosome I Chromosome II	1080283–81164 2811530–12474 1873235–74092
*fliJ, fliH, fliG, fliF, fliE, fliM, fliN, fliP, fliQ, fliR, flhB, flhA, fliS, fliK, flgK, flgI, flgG, flgF, flgE, flgD, flgC, flgB, flgA, flgM*	Flagella	Chromosome II	1851413–07504
*cheX, cheY, cheA, cheW, cheV, cheR*	Chemotaxis protein	Chromosome I	330969–12985
**IRON UPTAKE**			
*hutZ, hutW, hutR*	Heme receptors	Chromosome II	1609317–54514
**SECRETION SYSTEM**			
*tssM, tssL, tssK, tssJ, fha, tssH, tssG, tssF, tssE, tssC, tssB, tssD, tssA, pAAR, tssI*	Type VI secretion protein	Chromosome I	1649571–72337
*tssH, tssD, tssI, ppkA, tssA, tssB, tssC, tssE, tssG, asnC, tssM, tssA, fha, tssJ, tssK, tssL, tssI fha, tssJ, tssK, tssL, tssM, tagF, pppA, tssA, tssB, tssC, tagJ, tssE, tssF, tssG, tssH, tssD, tssI, pAAR*	Type VI secretion protein	Chromosome II	850171–1172 936359–9932
*exsA, vscN, vsCC, vscQ, pscR, vscR, vscS, vsrD, lcrD, pcrD, vopD, vopB, vcrH, vcrG, vcrR, vscY,vscX, sycN, vopN, vscO, vscT, vopQ, vecA, vopS, vscL, vscJ, vscI, vscG, vscF, vscD, vscB, exsD, exsC*	Type III secretion protein	Chromosome I	2343847–69758
**TOXIN**			
*tlh*	Thermolabile hemolysin	Chromosome II	1935326–36582

Thirty-seven genes in *V. campbellii* LMB29 were observed showing >45% identity to well-characterized antimicrobial resistance genes in reference databases Resfinder and CARD (Table 2). Multiple genes encoding aminoglycoside phosphotransferase [*strB, strA* and *APH(3*″*)-Ib*], tetracycline resistance genes [*tet(A), tet35* and *tet34*] and *blaACARB*-*18* (a beta-lactamase coding gene) were predicted in the LMB29 genome. These results are consistent with our antibiotic susceptibility assay data for strain LMB29 by showing its resistance to ampicillin, amoxicillin, rifampicin, tetracycline, streptomycin, oxacillin, lincomycin, teicoplanin, and vancomycin, some of which are classified as human medically-important antibiotics.

**Table 2:** Antibiotic resistance genes detected in *V. campbellii* LMB29.

**Gene location**	**Gene**	**% Identity^a^**	**Description**	**Accession Number^a^**
plasmid1_orf00191	*sul2*	100.00	Sulfonamide resistant dihydropteroate synthase	KF152885.1
plasmid1_orf00193	*strB*	100.00	Aminoglycoside phosphotransferase	KP143090.1
plasmid1_orf00196	*tet(A)*	99.83	Tetracycline resistance MFS efflux pump	AY196695
chr1_orf02269	*tet35*	99.73	Tetracycline efflux pump	AF353562
plasmid1_orf00192	*strA*	99.63	Aminoglycoside phosphotransferase	KP143090.1
plasmid1_orf00004	*arr-9*	99.00	Rifampin ADPribosyl transferase	HE577629.1
chr1_orf00278	*APH(3″)-Ib*	95.97	Aminoglycoside phosphotransferase	M86701.1
chr1_orf00257	*crp*	95.24	Regulator that represses MdtEF multidrug efflux pump	NC_007779.1.12933934
chr1_orf00116 &chr1_orf00281	*VCD_001261*	94.91	Translation elongation factor Tu	CP001485.1
chr1_orf02843	*tet34*	92.86	Mg2+-dependent oxytetracycline resistance determinant	AB061440
chr1_orf00124	*rpoB*	85.02	Resistance to rifampicin	U00096.3
chr2_orf00599	*blaACARB-18*	83.93	Beta-lactamase	KJ934266.1
chr1_orf03098	*parE*	79.30	Fluoroquinolones resistance	NC_003197.1.1254704
chr1_orf03274	*ugd*	77.32	Polymyxin resistance	U00096.3
chr1_orf01308	*gyrA*	75.20	Fluoroquinolones resistance	U00096.3
chr1_orf03097	*parC*	69.59	Fluoroquinolones resistance	U00096.3
chr1_orf02330	*acrB*	64.51	Protein subunit of AcrA-AcrB-TolC multidrug efflux complex	DQ679966.1
chr1_orf01604	*FosC2*	62.12	Enzyme that phosphorylates fosfomycin	AB522969.1
chr1_orf00190	*cpxR*	60.70	Cefepime and chloramphenicol resistance	U00096.3
chr1_orf00627	*folP*	60.66	Sulfonamide resistance	U00096.3
chr2_orf00310	*mdtL*	60.25	Multidrug resistance efflux pump	U00096.3
chr2_orf02196	*qnrVC1*	59.72	Fluoroquinolones resistance	EU436855.2
chr1_orf01887	*mdtK*	56.63	Multidrug and toxic compound extrusions (MATE) transporter	U00096.3
chr1_orf02270	*hns*	56.62	Repressor of many RND-type multidrug exporters.	U00096.3
chr1_orf02321	*katG*	55.75	Catalase-peroxidases that catalyzes the activation of isoniazid	AL123456.3
chr2_orf00412	*soxR*	54.93	A sensory protein that leads to the expression many multidrug efflux pumps	U00096.3
chr1_orf00125	*rpoC*	54.09	Daptomycin resistance	NC_002952.2860170
chr2_orf00011	*catB9*	53.21	Chloramphenicol resistance	AF462019.1
chr1_orf03175	*dfrA3*	51.88	Trimethoprim resistant dihydrofolate	J03306
chr1_orf01596	*ABBFA_001145*	51.49	Multidrug resitance protein	NC_011595.7059027
chr1_orf02331	*smeD*	47.55	SmeDEF multidrug efflux complex	AJ252200.1
chr1_orf03103	*tolC*	46.59	Multidrug resistance efflux pump	FJ768952.1
chr1_orf03500	*mexI*	46.33	Multidrug resistance efflux pump	NC_002516.2.880346
chr2_orf00587	*mexF*	45.84	Multidrug resistance efflux pump	NC_002516.2.882884
chr1_orf00189	*cpxA*	45.18	Cefepime and chloramphenicol resistance	U00096.3
chr2_orf02092	*emrD*	45.11	Multidrug resistance efflux pump	FJ744595.1

a *Percent nucleotide identity and corresponding GenBank accession number for reference sequence*.

**Figure 6:**
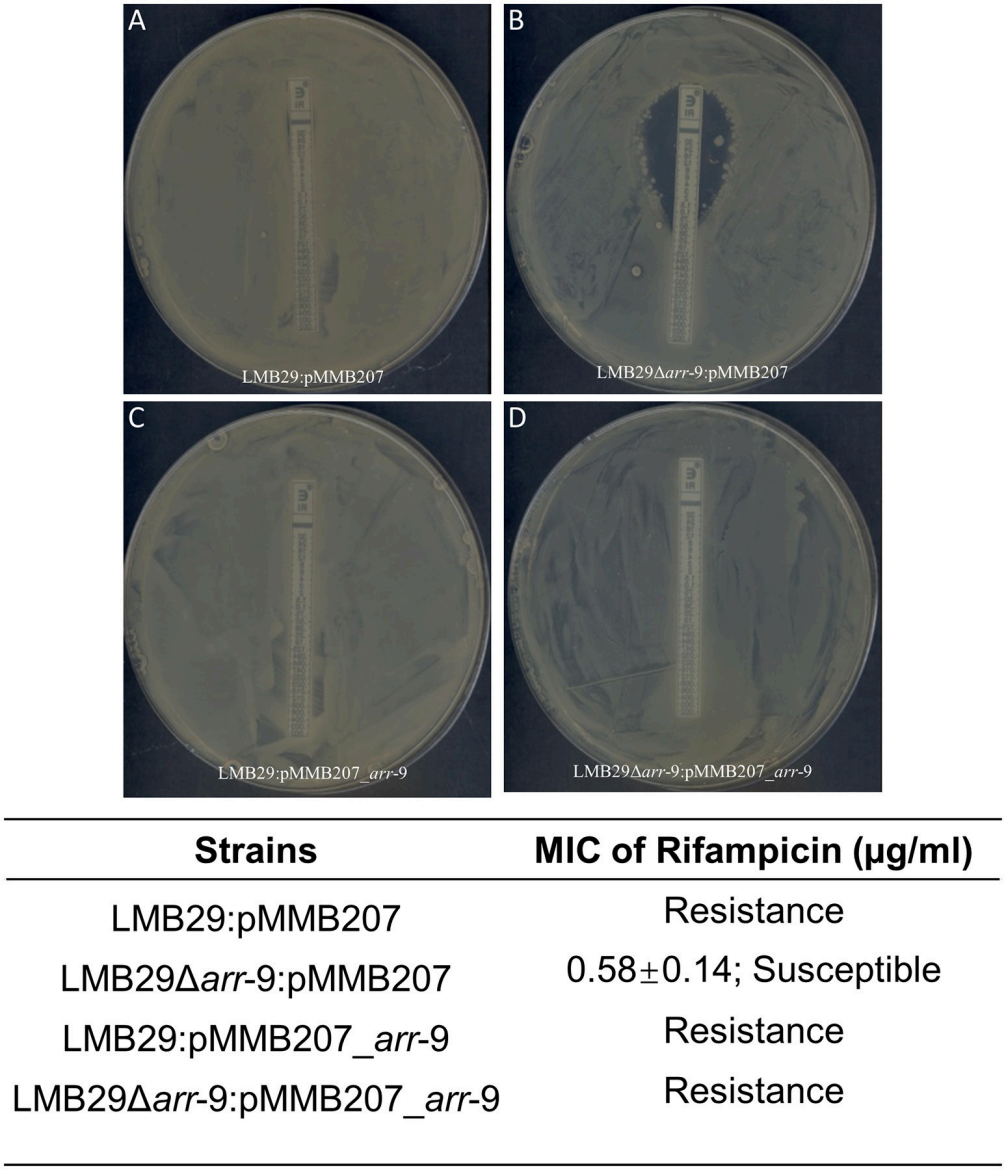
*arr*-9 is responsible for rifampicin resistance in *V. campbellii* LMB 29. The assay was performed in triplicates, and representative images were shown in **(A–D)**. MIC of rifampicin are expressed as means ± SEM from three independent experiments.

**Figure 7:**
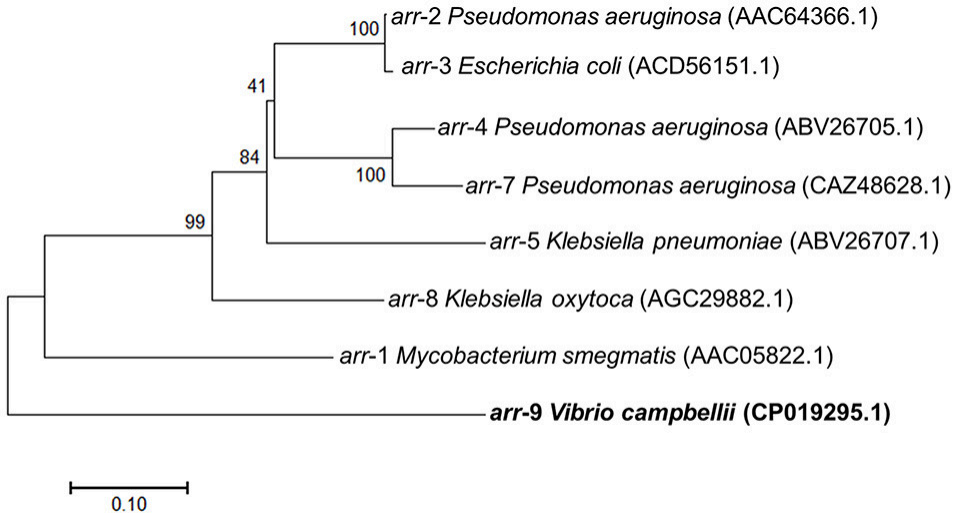
The phylogenetic tree of transmissible rifampicin resistance genes (*arr*-1–*arr*-9).

**Figure 8:**
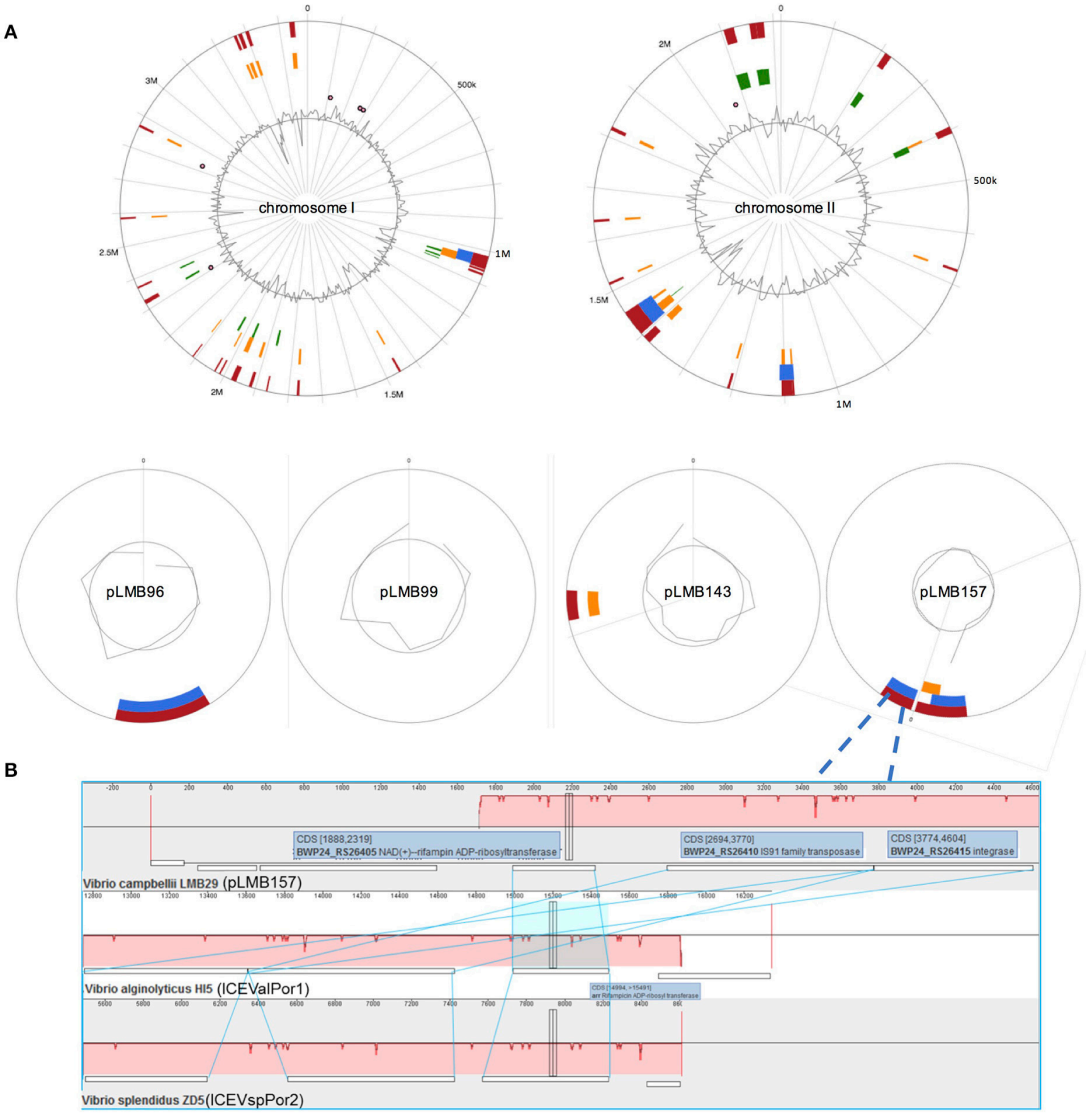
**(A)** Genomic islands (GI) prediction in *V. campbellii* LMB29 through IslandViewer 4. The predicted GIs are colored based on the prediction methods. Red indicates an integrated analysis, blue represents IslandPath-DIMOB prediction; orange represents SIGI-HMM and green color indicates IslandPick analysis. **(B)** The *arr*-9 gene cassette (*arr*-9 -transferase-integrase) alignments in *V. campbellii* LMB29 pLMB157, *V. alginolyticus* HI5 ICEValPor1, and *V. splendidus* ZD5 ICEVspPor2.

Moreover, in plasmid pLMB157, we named a gene *arr*-9 which shows 99% identity to a previously described rifampin ADP ribosyltransferase (Arr) (Rodriguez-Blanco et al., 2012). We demonstrated that the *arr*-9 is responsible for rifampin-resistance phenotype of LMB29 since the deletion mutant was showing susceptibility to rifampin (Figure 6B), and complementation of this gene successfully restored the resistance phenotype (Figure 6). We also constructed the phylogenetic tree for *arr*-9 with all previously reported rifampin ADP-ribosyltransferase and *arr*-9 shows a <10% identity to all other genes (Figure 7). This gene was originally reported in integrating conjugative elements (ICEs) in *Vibrio splendidus* and *Vibrio alginolyticus* (Rodriguez-Blanco et al., 2012), and the current study is the first report showing its presence in a mobilized plasmid (Figure 8B).

Genomic islands (GIs), regions of probable horizontal origin, were predicted in *V. campbellii* LMB29, and multiple GIs were identified in this strain with transferable potentials (Figure 8A; Supplementary Table 3). *arr*-9 was predicted sits on a 6,844 bp genomic island in pLMB157 which highlights its potential of horizontal transmission (Figure 8A). In addition to *arr*-9, two aminoglycoside O-phosphotransferase (*strA* and *strB*), one sulfonamide-resistant dihydropteroate synthase (*sul2*) and one tetracycline resistance MFS efflux pump gene [*tet(A)*] were predicted on another genomic island (7,797 bp) in plasmid pLMB157. These findings are consistent with bacterial adaptations to various environments by acquiring antimicrobial resistance.

## Discussion

**Figure 9:**
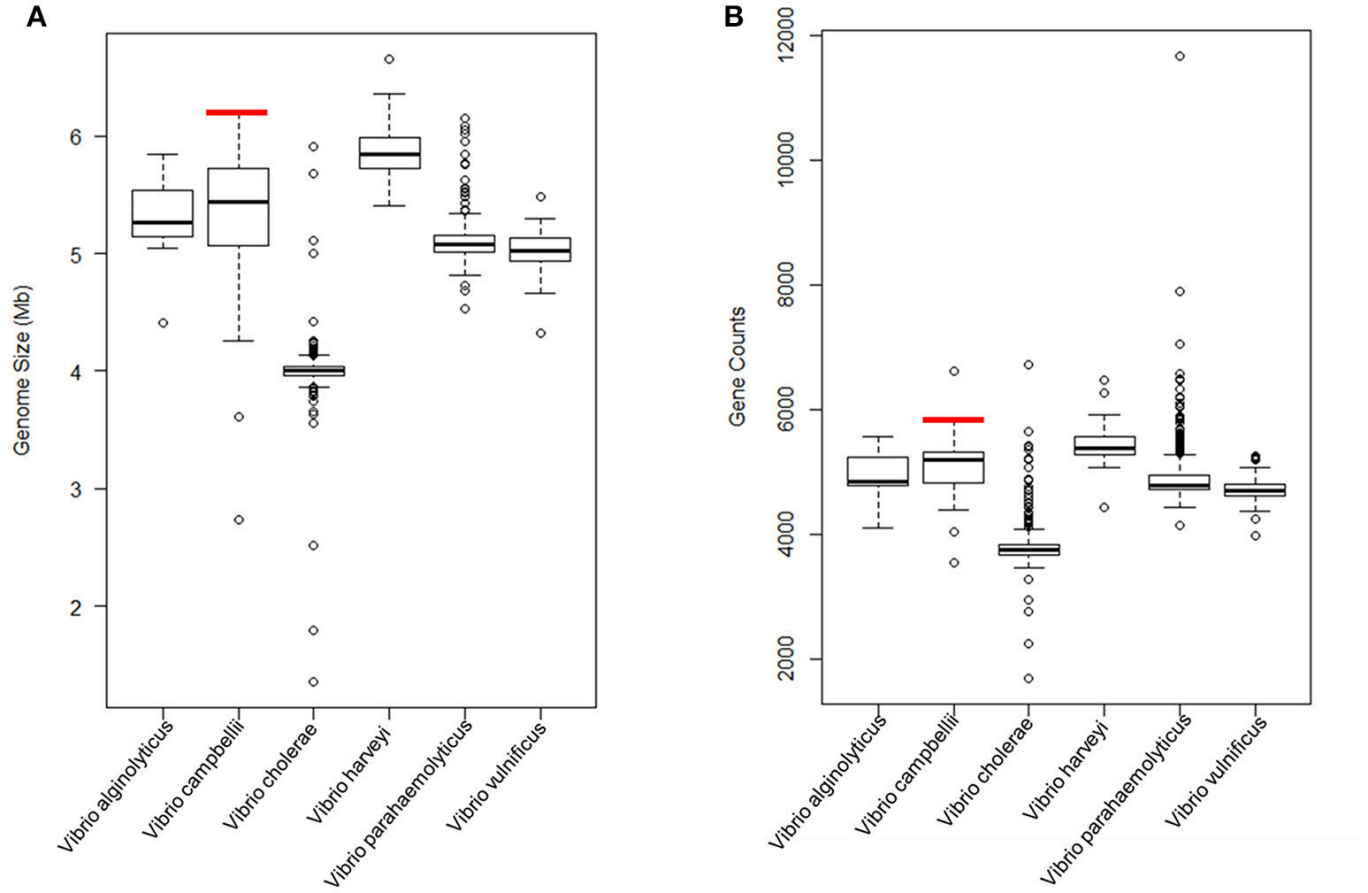
Genome size **(A)** and gene counts **(B)** comparisons between *V. campbellii* LMB29 and other *Vibrio* spp. (1,045 genomes). Red line indicates *V. campbellii* LMB29.

**Figure 10:**
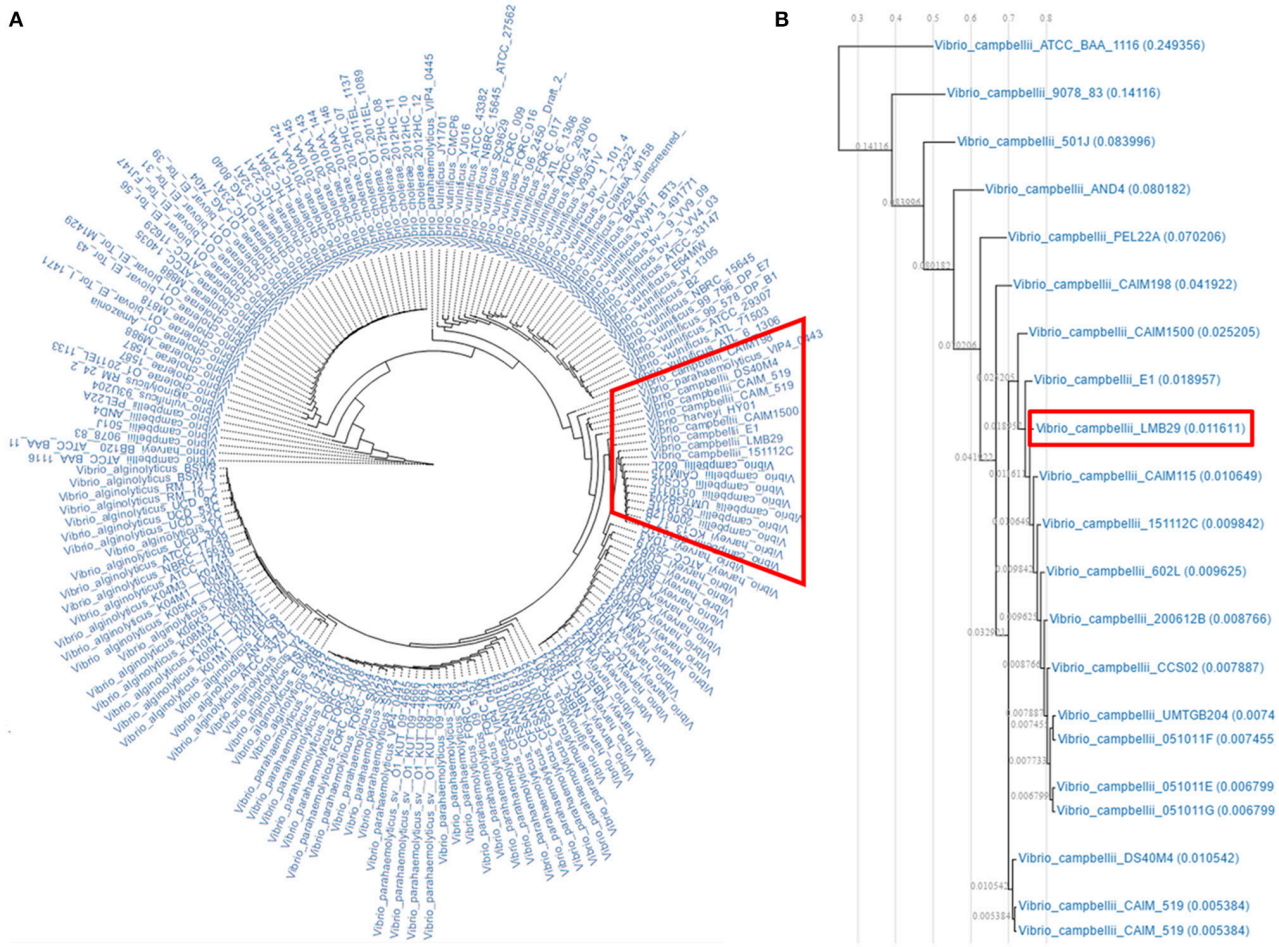
The predicted genome function comparisons between *V. campbellii* LMB29 and others. **(A)** Functional profile tree of *V. campbellii* LMB29 and other *Vibrio* spp. **(B)** The functional relationship of *V. campbellii* LMB29 to other *V. campbellii* strains.

The complete genome sequence of *V. campbellii* LMB29 was determined in this study, and the genome (6,283,706 bp) was assembled into two circular chromosomes and four plasmids. Compared with other members of this bacterial species (21 *V. campbellii* strains available in JGI IMG/MER database, Supplementary Table 1), LMB29 harbors a larger genome with 5,825 predicted genes (Figure 9). The four megaplasmids were believed to contribute to the larger genome size, but their detailed evolutionary benefits need to be further explored. We also assessed the functional profile of LMB29 and other *Vibrio* spp. based on the COG database, and genome clustering results was shown in hierarchical tress (Figure 10). In general, *V. campbellii* LMB29 shares similar function profile with other *V. campbellii* strains, but also includes some pathogenic species (e.g., *V. harveyi* HY01 and *V. parahaemolyticus* VIP4-0443) which indicates the pathogenesis of this strain (Figure 10). *V. campbellii* sits close to *V. harveyi* and *V. vulnificus* in the function profiling tree.

*Vibrio campbellii* LMB29 was isolated and studied because of its virulence to red drum in Shenzhen, China. Its cytotoxicity toward fish cells was assessed and confirmed by the LDH assay, and LMB29 infection was shown contributing to the DNA fragmentation (Figure 5). In addition, various virulence factors, two secretion systems (T3SS and T6SS) along with one thermolabile hemolysin encoding gene, *tlh*, were identified in this strain. These observations are consistent with its pathogenic phenotype which was involved in to serious fish disease in clinics.

*Vibrio* spp. are usually susceptible to most antimicrobials of veterinary and human significance (Elmahdi et al., 2016). While this *V. campbellii* LMB29 was showing a multidrug resistance phenotype to at least nine antibiotics we assessed including human medically-important drugs vancomycin and rifampicin. Rifampicin is one of the most potent and broad spectrum antibiotics which are widely used to against bacterial pathogens. Since its introduction in the medical practice, rifampicin has become a front-line drug for treating tuberculosis, leprosy and many other widespread diseases (Alifano et al., 2015). Resistance to rifampicin, a notable global health problem concern, is nearly always due to point mutations in the β-subunit of RNA polymerase in different bacterial species (Goldstein, 2014). However, no change was found in their RNA polymerases of several rifampicin-resistant strains of *Vibrio* species, including the *V. campbellii* LMB29, when compared to rifampicin-susceptible strains (data not shown). Interestingly, one gene (BWP24_RS26405), encoding a rifampin ADP-ribosyl transferase, was found in plasmid pLMB157 of *V. campbellii* LMB29. This gene, we named *arr-9*, was originally reported in integrating conjugative elements (ICEVspPor2 and ICEValPor1) (Rodriguez-Blanco et al., 2012), and this current study is the first report showing its presence on a transmissible plasmid. We experimentally confirmed the gene function of *arr-9* by showing its knock-out mutant loses the resistance phenotype, and the phenotype was restored by overexpressing this gene (Figure 6). This gene *arr*-9 along with one IS91 family transposase and one integrase were predicted sit on one genomic island which could be easily transmitted, and its dissemination will further compromise the *Vibrio* infections which will also limit our treatment options (Figure 8).

Overall, we present here the first complete genome of one predominant diseases-causing *V. campbellii* LMB29. The genome was queried as an effort to study its antimicrobial resistance and to identify potential virulence factors. Multiple resistance genes and virulence factors were predicted in this strain, and we experimentally confirmed its virulence to fish cells and the function of one rifampin ADP-ribosyl transferase encoding gene, *arr*-9. The high quality complete genome sequences generated in this study will form an important basis for further studies that will lead to a deeper understanding of the molecular mechanisms of *Vibrio* pathogenesis, thereby improving seafood quality and reducing economic loss.

## Data access

The complete genome sequence of *Vibrio campbellii* LMB29 has been deposited in GenBank under the accession numbers CP019293 (Chromosome I), CP019294 (Chromosome II), CP019295 (pLMB157), CP019296 (pLMB143), CP019297 (pLMB99), CP019298 (pLMB96).

## Author contributions

JL and ZZ conceived and designed the study, analyzed the data, and wrote the manuscript; JL, YD, YL, CW, PL, and YS performed the experiments and DNA preparation; PL and CH contributed the reagents. All authors read and approved the manuscript for publication.

### Conflict of interest statement

The authors declare that the research was conducted in the absence of any commercial or financial relationships that could be construed as a potential conflict of interest.
