# Supplementary material for: Complete Genome Sequence of Vibrio campbellii LMB 29 Isolated from Red Drum with Four Native Megaplasmids
Source: Front Microbiol. 2017 Oct 23;8:2035. doi: 10.3389/fmicb.2017.02035 (PMC5660062; doi:10.3389/fmicb.2017.02035)
Supplement: Supplementary file 2 [file Table2.DOCX]

**Supplemental Table 2. Primers used in this study.**

| **Primers** | **Sequence (5'-3')** |
| --- | --- |
| pSW7848-F | GTCTGATTCGTTACCAATTATGACAAC |
| pSW7848-R | GAATTCGATATCAAGCTTATCGATAC |
| Arr9-up-F | AAGCTTGATATCGAATTCGGTCAACTCGGCATGACTTG |
| Arr9-up-R | CCGACTTGATCTTTTTTCCTAGTTATGGTTTTTATACTAAATATTG |
| Arr9-down-F | GAAAAAAGATCAAGTCGGTGCACATGAC |
| Arr9-down-R | TTGGTAACGAATCAGACACCGCTTCGTTATATTTGCAC |
| Arr9-outer-F | GATGAAGATAAATCGGATAACGTCAT |
| Arr9-outer-R | AGAAATGAGTTAGAGCCGACC |
| pMMB207-F | AGAAGCGGTCTGATAAAACAGAATTTGC |
| pMMB207-R | GCGCAACGCAATTAATGTAAGTTAG |
| Arr9-F | CATTAATTGCGTTGCGCACAATGATGACGTCAAACCATTTAC |
| Arr9-R | TTTATCAGACCGCTTCTGATCCCCAATGACGTGGAC |
